# Antifungal Susceptibility Profile of Clinical and Environmental Isolates of Aspergillus Species From a Tertiary Care Center in North India

**DOI:** 10.7759/cureus.54586

**Published:** 2024-02-20

**Authors:** Manharpreet Kaur, Nidhi Singla, Deepak Aggarwal, Reetu Kundu, Neelam Gulati, Mani Bhushan Kumar, Satinder Gombar, Jagdish Chander

**Affiliations:** 1 Microbiology, Postgraduate Institute of Medical Education and Research, Chandigarh, Chandigarh, IND; 2 Microbiology, Government Medical College, Chandigarh, Chandigarh, IND; 3 Pulmonary Medicine, Government Medical College and Hospital, Chandigarh, Chandigarh, IND; 4 Pathology, Postgraduate Institute of Medical Education and Research, Chandigarh, Chandigarh, IND; 5 Clinical Microbiology, Government Medical College and Hospital, Chandigarh, Chandigarh, IND; 6 Anesthesia and Intensive Care, Government Medical College and Hospital, Chandigarh, Chandigarh, IND

**Keywords:** caspofungin, aspergillus fumigatus, aspergillus flavus, antifungal susceptibility, amphotericin b

## Abstract

Introduction: *Aspergillus *species are ubiquitously found in the environment worldwide and are important causative agents for infection. Drug resistance among *Aspergillus *species is emerging, hence the present study was undertaken to look for antifungal susceptibility profiles of clinical and environmental isolates of *Aspergillus *species.

Materials and methods: During the period from January 2018 to June 2019, a total of 102 *Aspergillus *isolates (40 clinical, 40 hospital, and 22 community environment) were tested for antifungal susceptibility testing for determination of minimum inhibitory concentration (MIC)/minimum effective concentration (MEC) as per Clinical and Laboratory Standards Institute (CLSI) M38-A3 method for itraconazole, voriconazole, amphotericin B, and caspofungin.

Results: Out of these 102 *Aspergillus *isolates, *A. flavus *was the most common species present. *Aspergillus *species were found to have low MIC values to azoles such as itraconazole and voriconazole except for one clinical isolate, which showed a MIC value of 2 μg/ml to voriconazole. Two isolates were non-wild-type for amphotericin B, but all isolates were wild-type for caspofungin.

Conclusion: Antifungal susceptibility testing among clinical *Aspergillus *isolates and environmental surveillance studies in view of emerging drug resistance should be undertaken at a larger scale.

## Introduction

*Aspergillus *species primarily cause pulmonary infection with the involvement of other body sites like paranasal sinuses and cutaneous tissue. Infection is usually airborne and depending upon the severity of infection and species involved, it can cause fatal consequences [1]. The most commonly encountered *Aspergillus* species are *Aspergillus flavus*, *Aspergillus ​​​​​​fumigatus*, and *Aspergillus niger*. A few other species that are also frequently isolated nowadays are *Aspergillus ​​​​​​terreus, Aspergillus ​​​​​​glaucus, *and *Aspergillus ​​​​​nidulans* [2]. The azole group of drugs shows species- and strain-dependent fungicidal activity against *Aspergillus *species [3]. Voriconazole is the most commonly used drug in case of invasive aspergillosis [4]. Although *Aspergillus *species are generally susceptible to various compounds, intrinsic and acquired resistance has been documented against the azoles [5]. Surveillance studies indicate that the prevalence of resistance varies widely among countries. Around the world, in France, India, Japan, China, Denmark, Spain, Switzerland, Norway, and Germany, resistance rates vary widely across medical centers, with some studies showing high resistance rates of up to 5% and others showing rates even lower than 1% [6-10]. Pesticide use in agriculture is also a contributing factor to the development of drug resistance and cross-resistance to the agricultural triazoles has been reported [11]. European environmental surveys reported azole-resistant Aspergillus fumigatus isolates in up to 12% of Dutch and 8% of Danish soil samples [12,13]. Infections by *Aspergilli *are mostly from the environment via inhalation; so, the presence of azole resistance in the environmental strains can be an important factor for the failure of azole therapy. Hence, the present study was planned to determine the antifungal susceptibility in *Aspergillus *isolates, both clinical and environmental, as a primary step toward this issue in our geographic settings (North India).

A part of this manuscript won the outstanding poster award at 9th Advances against *Aspergillosis *and *Mucormycosis *held at Lugano, Switzerland, on February 27 to 29, 2020, and Second Prize in an oral presentation at North West Microcon 2019 held at Pandit Bhagwat Dayal Sharma Post Graduate Institute of Medical Sciences (PGIMS) in Rohtak on November 16, 2019.

## Materials and methods

Clinical isolates

Between January 2018 and June 2019, a total of 10,541 clinical samples from patients suspected of having fungal infections were received in the mycology laboratory and processed as per the standard mycological techniques. Potassium hydroxide (KOH) wet mount was prepared for direct examination, and samples were inoculated in two tubes on Sabouraud dextrose agar (SDA) with antibiotics and without actidione for culture. The tubes were incubated at 37ºC and 25ºC, respectively. The culture tubes were examined daily for growth for one week and twice weekly for another three weeks. If growth was positive, lactophenol cotton blue mount was made, and genus and species identification was done morphologically. A detailed case history, examination, and other relevant workup were done for all the patients with positive clinical samples. The work was duly approved by the Institutional Ethics Committee letter issued dated December 8, 2017.

Environmental isolates

In the second part of the study, a total of 60 samples from the hospital environment, such as air samples from hospital wards and intensive care unit (ICU), as well as 60 samples from the community environment, such as flower pots, rice paddy fields, soil admixed with bird droppings, and soil from gardens, were taken.

For Sampling From the Hospital Environment

The settle plate method was used as follows: A set of two Petri dishes containing blood agar and SDA media were kept in a designated area after removing the culture plate's lid. The plates were left in the area for the settlement of environmental dust for 30 minutes. After that, the plates were collected and further incubated at 25ºC for seven days. Then the fungal culture was examined daily for any growth of fungi [14].

For Soil Samples From the Community Environment

The soil plate method was used as follows: A minute quantity of soil sample was taken from various locations in the community with the help of a sterile needle tip and was placed on a drop of sterile water in the Petri dish and mixed. Then 15-20 ml of cooled molten SDA was dispensed into the Petri dish and gently rotated for even dispersion of soil into the medium. Then the plates were observed for seven days daily for the growth of fungi [15].

All types of fungal growth obtained were identified by standard mycological methods. If species identification was not possible from isolated growth, slide culture was put on cornmeal agar/oatmeal agar to enhance sporulation for further identification of the fungal isolates.

Among these, 40 samples from the hospital environment and 22 samples from the community environment came out to be positive for the growth of *Aspergillus* species. All the clinical strains (40) and environmental strains (62) were included further for antifungal susceptibility testing as per Clinical and Laboratory Standards Institute (CLSI) M38-A3 [16]. *A. flavus* 204304 was used as the control strain. A fresh subculture of the isolates was done on potato dextrose agar, and a final concentration of conidial suspension in the range of 0.09-0.13 for *Aspergillus* species at an optical density of 530 nm was chosen as inoculum. The drug concentrations tested for amphotericin B (AmpB), itraconazole, voriconazole, and caspofungin ranged from 0.0313 to 16 μg/mL. They were prepared in microwell plates using Roswell Park Memorial Institute (RPMI) 1640 medium. Plates were incubated at 37ºC for 48 hours (24 hours for caspofungin) before the final reading was taken.

Minimum inhibitory concentration (MIC) for azoles and amphotericin B was taken as the lowest concentration of an antimicrobial agent that causes a 100% reduction of visible growth of the isolate, while minimum effective concentration (MEC) is the term used for echinocandins (caspofungin) and is taken as the lowest concentration of an antimicrobial agent that leads to the growth of small, rounded, compact hyphal forms as compared to the hyphal growth seen in the growth control well [16]. Antifungal susceptibility testing results were analyzed for epidemiological cutoff values (ECV) according to CLSI M59 second edition [17]. The ECV is the MIC or MEC value that defines the upper limit of the wild-type (WT) distribution. It helps in differentiating between WT isolates and non-wild-type isolates. Wild isolates are without intrinsic or acquired resistance mechanisms, while non-WT isolates may have intrinsic or acquired resistance mechanisms.

## Results

Out of a total of 10,541 clinical samples received in the laboratory during this period, 521 (4.94%) samples were found to be positive for mycelial fungal growth. The present study included only 40 cases where the growth of *Aspergillus* isolates was considered significant, based on positive direct smear examination (presence of septate hyphae) and positive culture (*Aspergillus* species) with good clinical correlation. Forty samples from the hospital environment and 22 samples from the community environment were positive for *Aspergillus *species. Table 1 shows the MIC/MEC range, geometric mean (GM), MIC50/MEC50, and MIC/MEC90 of all the isolates, and Tables 2, 3 show the MIC/MEC distribution of different isolates according to the site of isolation.

**Table 1:** MIC/MEC distribution, GM, MIC/MEC 50, and MIC/MEC 90 of different species of Aspergillus and sites of origin ITZ: Itraconazole; VCZ: Voriconazole: AmpB: Amphotericin B; CAS: Caspofungin; GM: Geometric mean; HE: Hospital environment; CE: Community environment; MIC: Minimum inhibitory concentration; MEC: Minimum effective concentration.

	MIC/MEC range (µg/ml)			GM (µg/ml)			MIC 50 or MEC 50/MIC90 or MEC 90 (µg/ml)		
Drugs	Clinical	HE	CE	Clinical	HE	CE	Clinical	HE	CE
	A. flavus								
ITZ	0.03-0.5	0.03-0.5	0.03-0.5	0.0544	0.075	0.0544	0.0312/0.125	0.06/0.25	0.0312/0.125
VCZ	0.125-2	0.03-1	0.125-2	0.535	0.339	0.535	0.5/1	0.5/0.5	0.5/1
AmpB	0.125-8	0.5-4	0.125-8	0.337	1.248	0.337	0.25/1	1/2	0.25/1
CAS	0.03-0.12	0.03-0.12	0.03-0.12	0.048	0.0494	0.048	0.06/0.06	0.06/0.06	0.06/0.06
	A. fumigatus								
ITZ	0.03-1	-	0.12-0.5	0.125	-	0.314	0.062/1	-	0.5/0.5
VCZ	0.03-0.5	-	0.12-0.25	0.125	-	0.198	0.125/0.5	-	0.25/0.25
AmpB	0.03-4	-	1-2	0.198	-	1.587	0.125/4	-	2/2
CAS	0.03-0.12	-	0.06-0.12	0.055	-	0.075	0.06/0.12	-	0.06/0.12
	A. niger								
ITZ	-	0.03-0.5	0.03-0.5	-	0.150	0.189	-	0.25/0.25	0.0312/0.125
VCZ	-	0.03-1	0.125-2	-	0.217	0.330	-	0.25/0.5	0.5/1
AmpB	-	0.25-2	0.125-8	-	0.415	0.524	-	0.25/1	0.25/1
CAS	-	0.03-0.06	0.03-0.12	-	0.047	0.057	-	0.06/0.06	0.06/0.06

**Table 2:** Antifungal susceptibility profile for voriconazole and itraconazole of Aspergillus species from different sites of origin VCZ: Voriconazole; ITZ: Itraconazole; A.: *Aspergillus; *MIC: Minimum inhibitory concentration.

Site of origin	Isolates	0.03	0.06	0.12	0.25	0.5	1	2	4
		VCZ-MIC in µg/ml							
Clinical (9)	A. fumigatus	1	2	3	2	1	-	-	-
Community environment (3)	A. fumigatus	-	-	1	2	-	-	-	-
Clinical (30)	A. flavus	-	-	3	5	9	12	1	-
Hospital environment (25)	A. flavus	1	-	2	8	12	2	-	-
Community environment (4)	A. flavus		-	-	1	1	2	-	-
Hospital environment (15)	A. niger	2	-	4	3	5	1	-	-
Community environment (15)	A. niger		-	3	5	5	2	-	-
		ITZ-MIC in µg/ml							
Clinical (9)	A. fumigatus	4	1	1	-	-	3	-	-
Community environment (3)	A. fumigatus	-	2	1	-	-	-	-	-
Clinical (30)	A. flavus	15	10	2	2	1	-	-	-
Hospital environment (25)	A. flavus	5	14	2	2	2	-	-	-
Community environment (4)	A. flavus	-	-	-	1	1	2	-	-
Hospital environment (15)	A. niger	3	-	3	8	1	-	-	-
Community environment (15)	A. niger	-	3	4	5	2	1	-	-

**Table 3:** Antifungal susceptibility profile for amphotericin B and caspofungin of Aspergillus species from different sites of origin AmpB: Amphotericin B; CAS: Caspofungin; A.: *Aspergillus; *MIC: Minimum inhibitory concentration; MEC: Minimum effective concentration. One isolate of *A. candidus* MIC/MEC-AmpB (1 µg/ml), VCZ (0.06 µg/ml), ITZ (0.25 µg/ml), and CAS (0.12 µg/ml). Note: No isolates of *Aspergillus fumigatus* in hospital environment and *Aspergillus niger* in clinical samples were found.

Site of origin	Isolates	0.03	0.06	0.12	0.25	0.5	1	2	4	8
		AmpB-MIC in µg/ml								
Clinical (9)	A. fumigatus	1	3	2	-	1	-	1	1	-
Community environment (3)	A. fumigatus	-	-	-	-	-	1	2	-	-
Clinical (30)	A. flavus	-	-	4	16	6	3	-	-	1
Hospital environment (25)	A. flavus	-	-	-	-	4	10	10	1	-
Community environment (4)	A. flavus	-	-	-	-	1	-	2	1	-
Hospital environment (15)	A. niger	-	-	-	8	4	2	1	-	-
Community environment (15)	A. niger	-	-	-	5	5	4	1	-	-
		CAS-MEC in µg/ml								
Clinical (9)	A. fumigatus	3	4	2	-	-	-	-	-	-
Community environment (3)	A. fumigatus	-	2	1	-	-	-	-	-	-
Clinical (30)	A. flavus	10	19	1	-	-	-	-	-	-
Hospital environment (25)	A. flavus	9	14	2	-	-	-	-	-	-
Community environment (4)	A. flavus	-	1	3	-	-	-	-	-	-
Hospital environment (15)	A. niger	5	10	-	-	-	-	-	-	-
Community environment (15)	A. niger	3	10	2	-	-	-	-	-	-

Clinical isolates

Among the 40 clinical isolates, 30 (75%) isolates were *A. flavus*, nine (22.5%) were *A. fumigatus*, and one (2.5%) was *A. candidus*. These isolates were obtained from 21 (42.5%) patients with fungal sinusitis, 11 (27.5%) with pulmonary aspergillosis, and four (10%) with cutaneous aspergillosis. Three (7.5%) isolates were from ocular cases, and one (2.5%) was a case of cerebral aspergillosis.

All 30 isolates of* A. flavus* had MIC/MEC less than the ECV for itraconazole, voriconazole, and caspofungin which indicates the WT. One strain was found to have a high MIC value (8 μg/ml) to amphotericin B indicating a non-WT. One strain had a value equal to ECV for voriconazole.

Among *A. fumigatus *(9), most of the strains were determined to be WT for itraconazole, voriconazole, and caspofungin. However, among these isolates, three exhibited a MIC for itraconazole that was equal to the ECV. Additionally, one isolate (11.11%) demonstrated a high MIC value of 4 μg/ml for amphotericin B, indicating a non-WT. One strain had a value of 2 μg/ml. The MIC value of *A. candidus* isolate is listed in Table 3.

Isolates from the hospital environment

In a hospital environment, among 60 samples collected, 40 (66.66%) samples grew *Aspergillus *species. Among the 40 isolates, 25 (62.5%) were *A. flavus* and 15 (37.5%) were *A. niger*. It is clearly significant that although the presence of fungal spores of *Aspergillus* in the hospital environment is high, no *A. fumigatus* was isolated.

All isolates of *A. flavus* and *A. niger* were found to have low MIC for voriconazole, itraconazole, and caspofungin. While one isolate of *A. flavus* had a high MIC value of 4 µg/ml for amphotericin B, 10 isolates had a MIC value of 2 µg/ml. One isolate of *A. niger *also* *had a MIC value equal to 2 µg/ml (Table 3).

Isolates from the community environment

In the community environment, among 60 samples, 22 (36.66%) samples grew *Aspergillus* species. Out of these, 15 (68.18%) were *Aspergillus niger*, four (18.18%) were *Aspergillus flavus*, and three (13.63%) were *A.* *fumigatus*. All species of *Aspergillus* from the community environment had low MIC values for voriconazole, itraconazole, and caspofungin. However, one isolate of *A. flavus *had MIC of 4 µg/ml, one isolate of *A. niger*, and two isolates of both *A. fumigatus* and *A. flavus *had MIC of 2 µg/ml for amphotericin B (Table 3).

## Discussion

In developing countries, *A. flavus *has been reported to be a more prevalent species causing aspergillosis, whereas in developed countries, *A. fumigatus* is a more common species [18]. Similarly, in the present study, *A. flavus *was more commonly isolated. It is speculated that *A. flavus* survives better in hot and arid conditions of the Asia and Middle East regions [18]. Drug resistance is emerging in *Aspergillus* isolates, so determining the MICs of antifungals against various isolates of *Aspergillus* species is highly valuable in guiding therapy. The susceptibility of the isolates depends on the type of species as well as the nature and concentration of the drug. The presence of cryptic species within species complexes with different resistance profiles is further changing the scenario.

Triazoles such as itraconazole, posaconazole, and especially voriconazole are usually the antifungals of choice used as effective drugs in the treatment of different clinical forms of aspergillosis. Since 1990, studies have reported the acquired resistance of *Aspergillus* to azoles [8]. In 1997, azole resistance was reported for the first time in two patients from the USA [19]. Since then, its frequency has gradually been on the rise, with maximum reports from the Netherlands. The main reason could be active work being done to detect the resistance, in this area, by eminent mycologists. Azole drugs act as competitive cyp51 inhibitors. So azole resistance involves a mutation in the cyp51 gene, which inhibits drug binding [20]. In the present study, one strain (2.5%) of *A. flavus*, isolated from sputum, was found to have a MIC value equal to ECV for voriconazole. The strain had been isolated from a male patient, a farmer by occupation, presenting with a history of cough, shortness of breath, and on-and-off fever for the last three years. The patient could not survive and died of type I respiratory failure as per the records available.

Previously, a study done by Paul et al. had reported six (5%) clinical isolates with voriconazole MIC greater than the ECV. That study proposes the possible role of multidrug efflux pumps, especially that of Cdr1B overexpression, in contributing to azole resistance in *A. flavus* [20]. Resistance in *Aspergillus* to azoles means increased chances of therapeutic failure, with increased hospital stay, increased hospital charges, and more morbidity and mortality. Overall, most of the strains in our study were WT for azoles tested.

Polyene compounds, i.e., amphotericin B (AmpB) and its lipid formulations, target ergosterol in the cell membrane and are not the drug of choice for *Aspergillus*, especially *A. flavus* which is considered intrinsically resistant to polyenes [18]. However, if there is resistance to azoles, AmpB becomes a preferred choice, so it is important to know the sensitivity profile of isolated *Aspergillus* strains to AmpB. An extensive review by Fakhim et al. has reported that 14.9% of *A. flavus*, 5.2% of *A. niger*, and 2.01% of *A. fumigatus* have AmpB resistance [21]. In the present study, one isolate (2.5%) of *A. fumigatus* and one isolate (2.5%) of *A. flavus* had MIC values of 4 and 8 µg/ml, respectively. These values indicate that these isolates are non-WT isolates. Table 3 shows that many isolates of *Aspergillus* species had MIC value of 2 µg/ml for AmpB. Notably, the *A. flavus* isolate obtained from the hospital environment exhibited a high GM of 1.248, and most isolates (84%) had MIC ≥ 1 µg/ml.This raises concern regarding the emerging drug resistance in *Aspergillus* species to AmpB. In one of the studies, it was reported that patients harboring strains having Amphotericin B with MIC > 2 μg/ml had higher mortality than those with MIC < 2 μg/ml [22]. In studies from Asian countries, a comparatively high resistance has also been reported in *A. niger* to amphotericin B [21].

Echinocandins (ECs) namely caspofungin, anidulafungin, and micafungin target 1,3-β-D-glucan synthesis in the cell wall. In the present study, all clinical samples exhibited lower MEC values than the established ECV values for caspofungin indicating WT strains. Similarly, environmental strains also had low MEC values. In other studies, EC resistance in *Aspergillus* has been reported to be associated with a mutation in the *fks1 *gene just like *Candida* species, a modification in enzyme glucan synthase, and adaptive mechanisms like tolerance due to epigenetic effect [23,24]. Presently, ECs have been used as a salvage therapy for invasive aspergillosis; however, they are speculated to take up a more central role in the treatment of invasive aspergillosis considering the scenario of emerging resistance to azoles among *Aspergillus*.

In clinical isolates, 20 patients had either prior exposure to antifungal drugs or were on antifungal therapy of azoles. Fourteen patients were on itraconazole, four were on voriconazole, and two were on both itraconazole and voriconazole. Prolonged azole prophylaxis/therapy among patients can be a reason for the development of drug resistance in *Aspergillus *[25].

CLSI has established the ECV to differentiate wild- and non-wild-type strains in the case of *Aspergillus*. However, the European Committee for Antimicrobial Susceptibility Testing (EUCAST) has validated breakpoints for some species of *Aspergillus *[26]. The introduction of azole screen agar [27] is definitely a welcome move especially for laboratories in resource-poor settings or peripheral centers as MICs testing via microbroth dilution method is technically demanding, and commercial products like readymade antifungal susceptibility testing (AFST) plates can be a financial burden.

Pan-azole-resistant strains have also been reported in the literature in azole-naive patients [28]. However, as the particular allele was present in unrelated patients, it was concluded that the origin of such strains was possibly from the environment. We tried taking a history from local gardeners regarding the pesticides/manure used by them in the gardens, but nothing conclusive could be derived. There is frequent change in the material or product provided to them as a part of government supply to be used as pesticides. It is important to detect the environmental presence of drug resistance in *Aspergilli *as the spores can contaminate the patient's surroundings, whether in the hospital or the community, and can lead to serious superadded fungal infections in immunocompromised patients or can lead to nosocomial outbreaks. Snelders et al. found genetically similar strains with TR34/L98H alleles in patients and flower beds outside the hospital. The extensive use of azole fungicides in the animal industry and agriculture was analyzed to be responsible for the emergence of such strains [29]. The presence of azoles in soil not only causes soil contamination but also seeps into the water bodies and can cause air pollution, intensifying the azole pressure and exposure to aspergilli lurking in the environment via inhalation [30].

The present study had its limitations. The sample size was small, and genotypic identification of *Aspergillus* species could not be planned due to financial constraints. Similarly, it was not possible to identify the genetic mechanism responsible for higher MIC values or possible drug resistance among isolates.

## Conclusions

*A. flavus* was a common species isolated in clinical cases and hospital environments, whereas *A. niger *was common in soil samples from the community. Hospital environmental isolates displayed a tendency toward higher MIC. The laboratories are still struggling to include microbroth dilution methods as a routine procedure for antifungals, but it should not limit the institutions to take up studies, whenever possible, considering the need to generate sufficient baseline data as far as antifungal drug susceptibility is concerned. Environmental surveillance can be the key to analyze the current extent of drug resistance to further monitor and prevent the spread as well as to prepare the policy guidelines.
